# Effect of superfine grinding on the physicochemical properties of bulbs of *Fritillaria unibracteata* Hsiao et K.C. Hsia powder

**DOI:** 10.1002/fsn3.1203

**Published:** 2019-09-13

**Authors:** Cai‐xia Li, Ying‐ying Liu, Hai‐sheng Feng, Shi‐zhen Ma

**Affiliations:** ^1^ Northwest Institute of Plateau Biology Chinese Academy of Sciences Xining China; ^2^ Qinghai Provincial Key Laboratory of Qinghai‐Tibet Plateau Biological Resources Xining China

**Keywords:** *Fritillaria unibracteata*, particle size, powder, property, superfine grinding

## Abstract

This work aimed to determine the influence of superfine grinding on the physicochemical properties of bulbs of *Fritillaria unibracteata* Hsiao et K.C. Hsia (BFU) powder. For this purpose, fine powder (FP) and two superfine powders (SPs) were obtained via superfine and conventional grinding methods. The properties of different powders were studied and compared. Compared with FP, SPs exhibited higher values in terms of the angle of repose, swelling capacity, ethanol extraction yield, total alkaloid content, and imperialine content, while lower values in terms of particle size and bulk density. Especially, the total alkaloid content of SP‐I increased by 66.7%. Proper grinding is more conducive to reduce particle size and improve alkaloid content. FTIR analysis indicates that no new functional groups produced after superfine grinding. XRD analysis suggests that grinding treatment lead to decreases in the crystallinity. Therefore, superfine grinding displays immense potential in the BFU application.

## 1 INTRODUCTION

Bulbs of *Fritillaria unibracteata* Hsiao et K.C. Hsia (BFU) is a valuable health food and a well‐known traditional Chinese herb (State Pharmacopoeia Committee, 2015a). Specifically, BFU is mainly used to ease coughing and remove phlegm and is considered to exhibit more positive effects relative to other *Fritillaria* species (Zhou et al., 2017). Alkaloids were considered as the active ingredients of BFU that significantly inhibit cough frequency (Hao, Gu, Xiao, & Peng, 2013). Starch is the main component in BFU and accounts for approximately 70%–80% of the total biomass of *Fritillaria* (Gao, Fan, & Paek, 1999). Given that BFU are mainly obtained from its wild species, excessive harvesting of the plants has led to a decline in the species (Li, Dai, & Chen, 2009). Furthermore, increasing market demands resulted in the surging prices of BFU, thereby augmenting its overexploitation and exacerbating its scarcity (Li et al., 2013). Thus, it is necessary to explore an effective utilization method to increase bioavailability and saving BFU resources.

Superfine grinding technology is a useful technology to fabricate superfine powder with changed surface properties and leads to excellent characteristics that are absent in conventional powders (Zhang, Zhang, & Shrestha, 2005). Extant studies report on the superfine grinding of a few foods and traditional Chinese medicinal materials (Chen, Ai, & Huang, 2014; Tao et al., 2014; Xiao, Zhang, Fan, & Han, 2017). The decrease in powder particle size with superfine grinding increases the particle surface area and the breakdown of cell walls, increases particle extraction, and improves particle activity (Zhao et al., 2014, 2010). Thus, superfine grinding is potentially a good method to improve food material bioavailability and bioactivity. However, there is a paucity of information on the effects of superfine grinding on the physicochemical properties of BFU powder.

The aim of the present study involves investigating the application of superfine grinding technology on BFU powders. To accomplish this, BFU powders with different sizes were manufactured. Additionally, we investigated the effects of superfine grinding on the BFU powder properties including particle size distribution, morphology, angle of repose, bulk density, moisture absorption, swelling capacity, ethanol extraction yield, total alkaloid content, and the contents of isosteroidal alkaloids (peimissine, verticine, verticinone, and imperialine). Fourier transform infrared (FTIR) spectroscopy and X‐ray diffraction (XRD) characterizations of different BFU powders were also compared.

## 2 MATERIALS AND METHODS

### 2.1 Materials

Dry BFU were collected from Songpan County of Sichuan Province in October 2016, and stored at 4°C. Sipeimine standard (HPLC ≥ 98%) was procured from Beijing Solaibao Technology Co. LTD. Peimissine, verticine, verticinone, and imperialine standards were purchased from National Institutes for Food and Drug Control. HPLC grade solvents including methanol and acetonitrile were purchased from XinLanJing International Corporation. All other chemical reagents were purchased locally and were of analytical grade.

### 2.2 Sample preparation

#### 2.2.1 Fine powder

The BFU were set in an oven (DHG‐9070A) at 40°C for 4 hr. Subsequently, the dried BFU was subjected to conventional grinding using a high‐speed multi‐function grinder (SL‐500A) for 30 s. The type of milling was knife mill. The crushing time was 30 s, and the motor rated speed was 29344 *g*. The prepared powder was termed as BFU fine powder (FP).

#### 2.2.2 Superfine powder

A certain amount of FP (5.00 g in each tank) was placed into a vertical planetary ball mill (MITR‐TRXQM‐0.4L; Changsha Miqi Instrument Equipment Co., Ltd). The grinding tank was composed of agate and exhibited a capacity of 100 ml. The agate grinding balls with different diameters (Φ5, Φ8, and Φ10, mm) were used. Superfine powder I (SP‐I) was ground under the following conditions: grinding time corresponding to 1 hr, the weight ratio of ball to material corresponding to 12:1, and rotating speed corresponding to 6 *g*. Superfine powder II (SP‐II) was ground under the following conditions: grinding time corresponding to 3 hr, the weight ratio of ball to material corresponding to 20:1, and rotating speed corresponding to 12 *g*.

### 2.3 Particle size measurements

The particle size distribution was measured using a Mastersizer 2000 (Malvern Instruments, Malvern) at room temperature. Approximately 0.1 g of powder was dispersed in 10 ml of ultrapure water and shaken on a vortex shaker (DL‐SC05, Beijing Donglinchangsheng Biotechnology Co., Ltd) until the sample was fully homogeneous. The obtained suspension was added dropwise to the sample area containing approximately 800 ml of ultrapure water until the range of light obscuration was between 10% and 20%. Stirring speed of dispersion unit was kept at 1,800 rpm. A general‐purpose analysis model was used with particle refractive and absorption indices of 1.520 and 0.1, respectively, while the refractive index of water was 1.330. Particle size distribution was characterized via *D*_90_ and the span value [(*D*_90_ − *D*_10_)/*D*_50_] wherein *D*_10_, *D*_50_, and *D*_90_ values represent 10%, 50%, and 90%, respectively, cumulative volume percentiles of particles with diameters smaller than the value. Three measurements were performed for each sample.

### 2.4 Scanning electron microscope (SEM)

The BFU powder samples were spread on a conductive adhesive carbon tape that was pasted on a sample stub. The morphological characteristics of different powder samples were investigated using a SU8010 field emission scanning electron microscope (SEM; Hitachi).

### 2.5 Moisture absorption

Based on drug moisture absorption test guidelines in Pharmacopoeia (State Pharmacopoeia Committee, 2015b), the moisture absorptions of different BFU powders were determined.

### 2.6 Angle of repose and bulk density

The angle of repose and bulk density of BFU powders were measured using appropriate methods previously validated by Zhao, Yang, Gai, and Yang (2009).

The bulk density (g/ml) was the density including pores and interparticle voids. Three types of BFU powders were filled in a 10‐ml volumetric flask (*M*_1_) up to the mark and were weighed (*M*_2_) separately. The bulk density of BFU powder was calculated as follows:

$$\text{Bulk}\;\text{density}=(M_{2}-M_{1})/10.$$

where *M*_2_ was the total weight of the BFU powder and flask, and *M*_1_ was the weight of the flask only. The measurement of each sample was repeated five times.

The angle of repose was measured using the following steps. Firstly, one filler was fixed above graph paper so that the distance of the paper from the outlet of the filler (*H*) was 3 cm, and the filler was vertical to the paper. Then, the BFU powder was poured into the filler until the tip of the powder cone touched the outlet of the filler. The diameter (2*R*) of the cone was measured for each powder. The angle of repose (*θ*) was calculated as the following formula:

$$\theta =\text{arctg}\left(2R/H\right).$$

where *R* was the radius of the cone formed by the BFU powder, and *H* was the distance from the paper to the outlet of the filler. The measurement of each sample was repeated five times.

### 2.7 Swelling capacity

The swelling capacities of different BFU powders were measured by the determination method of swelling capacity in Pharmacopoeia (State Pharmacopoeia Committee, 2015b).

### 2.8 Determination of ethanol extraction yield

Based on the extract determination of *Bulbus fritillariae cirrhosae* in Pharmacopoeia (State Pharmacopoeia Committee, 2015a), the ethanol extraction yields of FP, SP‐I, and SP‐II were determined by the hot dipping method. The dilute ethanol solution was prepared by diluting ethanol (529 ml) with water to 1,000 ml. BFU powder (2 g) was macerated with 50 ml dilute ethanol solution for 1 hr. The solution was heated and then kept slightly boiling for 1 hr. The total weight of flask, powder, and solvent was weighed before heating and the lost weight was restored. And the solution was filtered. The filtrate (25 ml) was placed in a dry evaporating dish, dried on a water bath. Then, the evaporating dish was dried at 105°C for 3 hr, cooled in a desiccator for 30 min, and weighed quickly and accurately. The ethanol extraction yield was calculated based on the increased weight of the evaporating dish after drying.

### 2.9 Determination of total alkaloid content

The total alkaloid contents of FP, SP‐I, and SP‐II were measured by the content determination method for *Bulbus fritillariae cirrhosae* in Pharmacopoeia (State Pharmacopoeia Committee, 2015a).

### 2.10 Determination of major alkaloids

#### 2.10.1 Sample preparation

Individual BFU powder (2 g) was prealkalized with 4 ml ammonium hydroxide for 1 hr, and mixed with 40 ml solution of trichloromethane–methanol (4:1, V/V). The mixtures were refluxed in 80°C water bath for 2 hr, the lost weight was restored, and the mixtures were filtrated. The filtrate (10 ml) was transferred to an evaporating dish and evaporated to dryness. The obtained residues were resolved with 2 ml methanol, and the resultant extracts were directly subjected to analysis.

#### 2.10.2 Chromatographic conditions

The samples were analyzed by Dionex U3000 HPLC system (Thermo Fisher), equipped with a pump and a CORONA Ultra Detector. An Eclipse XDB C18 column (150 mm × 4.6 mm I.D., 5 μm, Agilent) was utilized at a column temperature of 30°C. The mobile phase consisted of acetonitrile–methanol–water (65:10:25, v/v) containing 0.08% triethylamine with a flow rate of 1.0 ml/min. The evaporation temperature for detector was set at 30°C. Nitrogen was used as the carrier gas at a flow rate of 0.2 ml/min with the pressure of a nebulizing gas of 0.4 MPa. An aliquot (20 μl) of sample was injected.

### 2.11 Fourier transform infrared spectroscopy (FTIR)

The FTIR spectra of FP, SP‐I, and SP‐II were collected via a Nicolet model 8700 spectrometer (Nicolet Instrument Corporation) in the wavenumber range of 400–4,000 cm^−1^ with a spectral resolution of 4 cm^−1^. Samples were diluted with KBr mixing powder and pressed into self‐supporting disks.

### 2.12 X‐ray diffraction (XRD)

In this study, X‐ray diffraction analysis was employed to detect the crystallinity and was conducted using an XRD‐6000 diffractometer (Shimadzu) in Bragg–Brentano geometry. The powder was placed in a glass sample holder. Subsequently, Cu Kα radiation was generated at 30 mA and 40 kV. Samples were scanned from 5° to 80° with a step size of 0.02°.

### 2.13 Statistical analyses

Results were reported in conjunction with the standard deviation (*SD*). The differences in the mean were calculated using Duncan's multiple range tests with a 95% confidence limit (*p* < .05) and SPSS statics 22.0 software.

## 3 RESULTS

### 3.1 Particle size distribution and morphology

**Figure 1:**
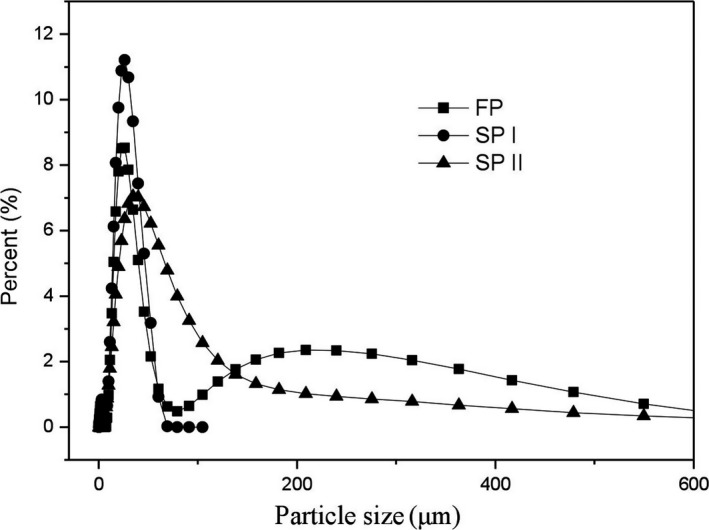
Particle size distributions of BFU powders. Particle size distributions of BFU powders shows particle size distribution curves of FP, SP‐I, and SP‐II. ■ represents FP (fine powder), ● represents SP‐I (superfine powder I), and ▲ represents SP‐II (superfine powder II)

Table 1 and Figure 1 show the particle size distributions of BFU powders. After proper grinding, SP‐I exhibited smaller particle size and smaller span value when compared to those of FP. Figure 1 also shows that the particles of FP were mainly distributed in the ranges of 0–80 μm and 80–600 μm and the particles of SP‐I exhibited a concentrated distribution in the range of 0–70 μm. This indicates that significant particle size reduction in SP‐I mainly resulted from the breakage of large particles and the formation of smaller particles, which was similar to the particle size reduction of microcrystalline cellulose via grinding (Zheng, Fu, Li, & Wu, 2018). After intense grinding, SP‐II exhibited smaller *D*_90_ and span value, but bigger *D*_10_ and *D*_50_ than FP. Additionally, the particles of SP‐II were mainly distributed in the range of 0–200 μm. This suggests that the breakage of large particles and the agglomeration of individual particles existed simultaneously for SP‐II, which was also similar to the breakage and agglomerative phenomena induced by prolonging grinding (Guzzo, Barros, & Tino, 2019; Zheng et al., 2018). Therefore, proper grinding significantly reduced the size of BFU powder and improved the particle size distribution.

**Table 1:** Particle sizes of BFU powders (Mean ± *SD*, *n* = 3)

	*D*_10_ (μm)	*D*_50_ (μm)	*D*_90_ (μm)	Span value
FP	13.85 ± 0.12b	30.78 ± 0.50b	271.95 ± 15.12c	8.38 ± 0.35c
SP‐I	11.26 ± 0.17a	25.69 ± 0.13a	45.15 ± 0.22a	1.32 ± 0.01a
SP‐II	16.00 ± 0.19c	42.34 ± 1.03c	154.02 ± 30.54b	3.25 ± 0.64b

Note

Different lowercase letters in the same column indicate significant difference among the different treatments at the 0.05 level.

**Figure 2:**
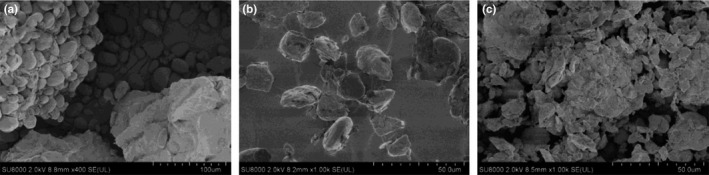
SEM images of (a) FP, (b) SP‐I, and (c) SP‐II. SEM images show the microstructures of BFU powders obtained by different methods. Image (a) corresponds to FP (fine powder, the particle size is 271.95 μm), image (b) corresponds to SP‐I (superfine powder I, the particle size is 45.15 μm), and image (c) corresponds to SP‐II (superfine powder II, the particle size is 154.02 μm)

The SEM images of different BFU powders are shown in Figure 2. For FP, particles with different sizes simultaneously existed with various particle shapes and a smooth particle surface. Various particle shapes were formed since the particles were squeezed during the growth process (Wang, 2006). For SP‐I, particle sizes were similar, particle shapes were irregular, particle surfaces were rough, and the particle edges were broken, indicating that the main effect of grinding was the breaking of BFU particles (Zheng et al., 2018). For SP‐II, broken particles and agglomeration of broken particles were observed. The SEM results were highly consistent with the particle size distribution results. The manufacturing of superfine powders was influenced by two counteracting processes: particle breakage and interparticle interaction (Austin & Bagga, 1981; Guzzo, Tino, & Santos, 2015). Particle breakage was considered to increase the specific surface area of the grinding product, and interparticle interactions governed by interfacial and surface properties were considered to reduce the specific surface area of grinding product (Guzzo et al., 2015). During grinding process, SP‐I and SP‐II underwent a morphological transformation under the effect of particle breakage and interparticle interaction (Fig. S1).

In particular, the agglomeration of superfine grinding particles was the typical behavior of powder system during grinding process (Opoczky, 1977). Huang, Lu, Li, and Tong (2007) reported that the formation of agglomeration in cassava starch by prolonging milling time was due to van der Waals force and electrostatic force. Zheng et al. (2018) also reported that the occurrence of agglomeration in microcrystalline cellulose by prolonging milling time was probably due to the high specific area and strong hydrogen bonds formed between the particles. With respect to the SP‐II, the agglomeration of broken particles was due to the mechanical force and interparticle interactions. The increases in the size and the width of the size distribution of SP‐II than SP‐I should be caused by agglomerations (Guzzo et al., 2019; Sundum, Szécsényi, & Kaewtatip, 2018). Therefore, intense grinding of SP‐II was unfavorable in terms of the particle size control of superfine BFU powder.

### 3.2 Physical properties of the BFU powder

The angle of repose reflects the change in the flowability of the powder (Ileleji & Zhou, 2008). As shown in Table 2, decreases in the particle size significantly increased the angle of repose of BFU powder, and this indicated that superfine powder exhibited decreased flowability. The result was in agreement with that stated in the reports (Huang, Dou, Li, & Wang, 2017; Zhao et al., 2014). According to Carr classification of flowability of powder based on repose angle (Al‐Hashemi & Al‐Amoudi, 2018; Riley & Hausner, 1970), SP‐I was cohesive, and SP‐II was fair to passable flow. Generally, angle of repose is related to the interparticle force, interaction force, and the gravity force (Raei, Asadi, Moussavi, & Ghadiri, 2015; Wang et al., 2010). When particles were small, the interparticle forces became dominant (Wang et al., 2010; Yang, Liu, Yang, & Cao, 2009). Lumay et al. (2012) also reported that the cohesion between the particles could affect the angle of repose when the particle sizes of flours were lower than 50 μm. Therefore, the decreased flowability was potential because the decrease in particle size increased the particle surface area per unit mass, providing a greater surface area for cohesive force to interact (Jan, Karde, Ghoroi, & Saxena, 2018). Superfine grinding treatment adversely affected the flowability of the BFU powder, which would result in blockage, unstable discharge, and subsequent stoppage of the equipment and processes (Garg, Mallick, Trinanes, & Berry, 2018).

**Table 2:** A angle of repose and bulk density of BFU powder (Mean ± *SD*, *n* = 5)

	Angle of repose (°)	Bulk density (g/ml)
FP	36.62 ± 2.79a	0.60 ± 0.00c
SP‐I	49.50 ± 1.20c	0.49 ± 0.00b
SP‐II	44.61 ± 3.44b	0.41 ± 0.00a

Note

Different lowercase letters in the same column indicate significant differences among the different treatments at the 0.05 level.

The bulk density is often used to characterize powder flow behavior, significantly affecting material handling and storage aspects (Vasilenko, Koynov, Glasser, & Muzzio, 2013; Zhao et al., 2013, 2010). The bulk density depends on multiple factors, such as particle size and shape, environmental conditions, and consolidating stress (Vasilenko et al., 2013). As shown in Table 2, the bulk density of FP (0.60 g/ml) with a *D*_90_ size of 271.95 μm exceeded that of SP‐I and SP‐II, and this was not in agreement with the results stated in the reports (Wu, Zhang, Wang, Mothibe, & Chen, 2012; Zhao et al., 2014). The low bulk density should be due to unusually large voidage between the particles (Shenoy et al., 2015; Zhao, Zhu, Zhang, & Tang, 2015). Pai and Okos (2013) demonstrated that for an assembly of small and large particles, as the proportion of smaller particles exceeded a certain value, bulk density decreased because of increased voidage between smaller particles. Mohammadi and Harnby (1997) and Liu, Lu, Poletto, Guo, and Gong (2017) also reported that when the particle size was small, interparticle forces were comparable with the particle weight and prevented particles from forming compact structures, which resulted in low‐density values and cohesive bulk powders. Additionally, the bulk density of SP‐II (0.41 g/ml) with a *D*_90_ size of 154.02 μm was lower than that of SP‐I. Furthermore, the BFU powder with lower bulk density will be adverse to the robustness of the process and the quality of final product, such as packing behavior, filling behavior in preparing tablets or capsule products (Vasilenko et al., 2013; Zhao et al., 2010).

The moisture absorption of material refers to its ability to absorb water under certain conditions of temperature and humidity, which can be used as a reference to select suitable pharmaceutical packaging and storage conditions (State Pharmacopoeia Committee, 2015b). A relatively large amount of moisture absorbed in powder significantly impacts the long‐term stability and performance of powder (Yu, Romeo, Cavallaro, & Chan, 2018). As shown in Table 3, there were no significant differences among BFU powders, thereby indicating that superfine grinding did not significantly influence the moisture absorption property of BFU powder. This was potentially related to the formation of a dense film on the BFU powder surface after certain moisture absorption, which blocked the diffusion path of moisture (Joshi & Petereit, 2013; Zhao et al., 2014). Additionally, the percentages of moisture absorption of BFU powders were more than 2%, but less than 15%, thereby indicating that BFU powder exhibited hygroscopicity (State Pharmacopoeia Committee, 2015b).

**Table 3:** Moisture adsorption, swelling capacity, ethanol extraction yield, and total alkaloid content of BFU powder (Mean ± *SD*, *n* = 3)

	Moisture adsorption (%)	Swelling capacity (ml/g)	Ethanol extraction yield (%)	Total alkaloid content (%)
FP	9.41 ± 2.16a	1.93 ± 0.15a	4.39 ± 0.25a	0.009 ± 0.001a
SP‐I	8.73 ± 0.92a	2.75 ± 0.30b	5.67 ± 0.26b	0.015 ± 0.001c
SP‐II	8.67 ± 2.47a	7.83 ± 0.24c	7.78 ± 0.90c	0.011 ± 0.001b

Note

Different lowercase letters in the same column indicate significant differences among the different treatments at the 0.05 level.

Swelling capacity is an important parameter that reflects the hydration ability. As shown in Table 3, the swelling capacities of SP‐I and SP‐II increased by 0.82 ml/g and 5.90 ml/g, respectively, when compared with the swelling capacity of FP (1.93 ml/g). Generally, it is difficult for water to enter the starch granules through the pores (Zhang et al., 2019). Given that increased surface area, polar groups, and other water‐binding sites were exposed to the surrounding water medium, the increased swelling capacities of SP‐I and SP‐II potentially related to the decreases in the size of BFU particles (Du, Zhu, & Xu, 2014). The stronger swelling capacity of SP‐II than that of SP‐I should be due to the fact that the active sites in starch granules increased as a result of the structure destruction of starch under intense grinding treatment (Hofmann et al., 2018; Pineda, Ojeda, Romero, Balu, & Luque, 2018). Previously, a similar pattern was observed for the greatly improved swelling capacity of acetylated starch by grinding (Zhang et al., 2019). Thus, superfine grinding treatment enhanced the swelling capacity of the BFU powder.

### 3.3 Ethanol extraction yield and alkaloid content

Plant extracts contain compounds that exhibit synergistic pharmacological effects (Yang et al., 2014), and thus, plant extraction yield is typically used as an index to measure the quality of a traditional Chinese herb. The ethanol extraction yields obtained from BFU powders are shown in Table 3. When compared to FP, the ethanol extraction yield of SP‐I increased by 29.2%. It is commonly accepted that smaller particles with higher specific surface enhanced the diffusion of chemical components (Huang, Li, & Wang, 2018). Similar results were observed for the extraction of superfine black tea powder (Xiao et al., 2017). Additionally, the ethanol extraction yield of SP‐II also exceeded that of SP‐I. As expected, superfine grinding significantly improved the ethanol extraction yield.

The total alkaloid contents of BFU powders are determined and shown in Table 3. Decreases in the *D*_90_ size of BFU powder from 271.95 μm to 45.15 μm significantly increased the total alkaloid content from 0.009% to 0.015%. When compared to FP, the total alkaloid content of SP‐I increased by 66.7%. Chen et al. (2014) also reported that the verticine dissolution rate of *Fritillaria thunbergii* Miq. superfine powder (*D*_50_ = 36.46 μm) was 59.5% higher than that of common powder (*D*_50_ = 158.65 μm). The increasing tendency of the total alkaloid content was observed with reductions in the particle size (Xiao et al., 2017). Research on the effect of superfine grinding on the properties of red grape pomace powders indicated that total polyphenolic content was improved by superfine grinding from 450.13 mg/100 g to 757.36 mg/100 g (Zhao et al., 2015). Zhao et al. (2009) also reported that the protein solubility of ginger powder increased with decreasing particle size from 300 to 8.34 μm. Xiao et al. (2017) attributed the higher caffeine yield of superfine black tea powder to the smaller particles with higher particle surface area. Li, Li, Liu, and Yin (2007) reported that the breakage of cell wall by superfine grinding also could greatly reduce the mass transfer resistance and enhance the diffusion of active ingredients. Therefore, the significant increase in alkaloids content of SP‐I can be integrated from the increase in particle surface area and the breakdown of the cell walls, indicating that the particle size was a key parameter associated with the release of active components (Astill, Birch, Dacombe, Humphrey, & Martin, 2001).

Furthermore, the contents of 4 alkaloids in different BFU powders were determined and are shown in Table 4. For the type A isosteroidal alkaloids, peimissine, verticine, and verticinone were found in almost all Beimu herbs; for the type B isosteroidal alkaloid, imperialine was detected in Chuan‐Beimu, Ping‐Beimu, Yi‐Beimu (Li, Li, Lin, Chan, & Ho, 2000). As shown in Table 4, there were no significant differences in the contents of verticine and verticinone among BFU powders. When compared to FP, the peimissine and imperialine contents of SP‐I increased significantly. When compared to FP, the peimissine content of SP‐II did not change significantly, and the imperialine content of SP‐II increased significantly. The increases in the peimissine and imperialine contents should be also attributed to the increase in particle surface area and the breakdown of the cell walls (Astill et al., 2001; Li et al., 2007). In addition, it can be seen from Table 4 that the contents of peimissine and imperialine were higher among 4 alkaloids in BFU powders. Zhou et al. (2008) reported that the content of peimissine was 68.92 μg/g, the contents of imperialine and verticine in *F. unibracteata* were at trace level, and the content of verticinone could not be detected. Zhou, Guo, Shen, Chen, and Qin (2014) found that the contents of peimissine, verticine, and verticinone in *F. unibracteata* were 21.5–24.1 μg/g, 65.8–81.4 μg/g, and at undetected level, separately. Wu et al. (2018) reported that the contents of imperialine and verticine & verticinone in *F. unibracteata* were 39.04 μg/g and 301.11 μg/g. These results confirmed the existence of peimissine and imperialine in BFU powders.

**Table 4:** Contents of isosteroidal alkaloids in BFU powders (Mean ± *SD*, *n* = 3)

	Peimissine (μg/g)	Imperialine (μg/g)	Verticine (μg/g)	Verticinone (μg/g)
FP	65.12 ± 2.67b	14.49 ± 0.54c	13.47 ± 1.83a	2.65 ± 0.20a
SP‐I	72.35 ± 3.70a	23.32 ± 0.92a	13.40 ± 2.57a	2.57 ± 0.19a
SP‐II	59.23 ± 2.96b	20.29 ± 0.73b	13.83 ± 1.93a	2.94 ± 0.37a

Note

Different lowercase letters in the same column indicate significant differences among the different treatments at the 0.05 level.

Therefore, the application of superfine grinding technology in BFU powder increased the extraction efficiency and improved the functional ingredient contents.

### 3.4 FTIR analysis

**Figure 3:**
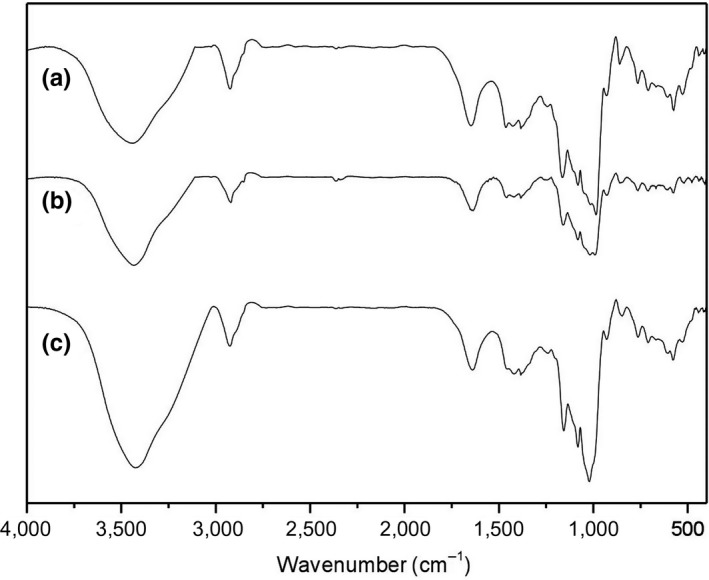
FTIR spectra of BFU powders: (a) FP; (b) SP‐I; (c) SP‐II. FTIR spectra show the chemical composition information of BFU powders obtained by different methods. Line (a) corresponds to FP (fine powder, the particle size is 271.95 μm), line (b) corresponds to SP‐I (superfine powder I, the particle size is 45.15 μm), and line (c) corresponds to SP‐II (superfine powder II, the particle size is 154.02 μm)

FTIR spectra of BFU powders are obtained and shown in Figure 3. For FP, the peaks at 3,440 and 2,900 cm^−1^ were due to the OH bonds and CH_2_ deformation, respectively (Dankarab, Haddaraha, Omara, Pujolàb, & Sepulcreb, 2018; Kacuráková & Mathlouthi, 1996). The peaks at 1,650 cm^−1^ were attributed to the absorbed H_2_O bending vibration (Kizil, Irudayaraj, & Seetharaman, 2002). The peaks at 1,163 cm^−1^ and 1,080 cm^−1^ were caused by C‐O stretching and anhydroglucose ring O–C stretching (Fang, Fowler, Tomkinson, & Hill, 2002; Warren, Gidley, & Flanagan, 2016). The bands at 1,047 cm^−1^ and 1,016 cm^−1^ were characteristic of the ordered and amorphous structure of starch (Liu, Ma, Yu, Shi, & Xue, 2011; Lopez‐Rubio, Flanagan, Shrestha, Gidley, & Gilbert, 2008).

As shown in Figure 3, SP‐I and SP‐II did not show any obvious new peaks. This indicates that no new functional groups were produced after superfine grinding. However, the intensities of peaks at the range of 1,000–1,650 cm^−1^ decreased after proper grinding, which should be mainly resulted from the particle size reduction (Tan et al., 2015; Zhao et al., 2013). After intense grinding, the intensities of peaks at 3,440 and 1,016 cm^−1^ increased, the peak at 1,047 cm^−1^ almost disappeared, and the intensity of peak at 985 cm^−1^ decreased. Liu et al. (2011) reported that the intensities of bands at 3,382 cm^−1^ in the treated maize starch spectra increased with the increase in grinding strength, which might be due to the transformation of hydrogen bond vibration mode caused by grinding treatment. The intensity change in bands at 985 cm^−1^, 1,016 cm^−1^, and 1,047 cm^−1^ should be due to the destroyed crystalline structure of starch in SP‐II (Ambigaipalan et al., 2011; Blaszczak, Valverde, & Fornal, 2005; Huang et al., 2007; Xie, Liu, & Cui, 2006). The crystalline structure of starch would be further demonstrated in X‐ray diffraction section.

### 3.5 XRD analysis

A starch granule exhibits a semicrystalline structure that comprises of crystalline and amorphous regions (Niu, Zhang, Jia, & Zhao, 2017). The ordered three‐dimensional structure of the amylopectin segment is associated with the crystallinity of starch granules (Bayer, Cagiao, & Calleja, 2006; Zobel, 1988).

To understand the changes in crystalline structure, the XRD curves of different BFU powders obtained after different processing treatments were obtained. With respect to the FP, an intense peak was observed at approximately 17° and a few peaks were observed at 15°, 20°, 22°, and 24° as well. The observed peaks of FP also indicate that the crystal type of BFU corresponded to type B, and this was consistent with Wang et al. (2007) and Martínez et al. (2019) results. After proper grinding, a few weak peaks were observed at 17°, 20°, and 22° for SP‐I. Furthermore, after intense grinding, SP‐II exhibited peaks at 20° and 22°. The XRD results indicate that the crystalline peak intensities were gradually reduced with enhancements in the grinding strength, which reflected the decrease in crystallinity (Martínez‐Bustos, López‐Soto, San Martín‐Martínez, Zazueta‐Morales, & Velez‐Medina, 2007). This observation was in accordance with the report about maize starch (Liu et al., 2011).

Crystallinity in starch is determined by the following: (a) crystal size, (b) percentage of crystalline regions, (c) orientation of the double helices within the crystalline domains, and (d) extent of interaction between double helices (Hoover & Ratnayake, 2002). According to Hebeish, El‐Rafie, El‐Sheikh, and El‐Naggar (2014), the reduction in starch crystallite size arising from the reduction in particle size during grinding process could result in a decrease and disappearance of diffraction peaks. This reason is that small crystallites may not produce enough detectable reflection intensities, especially for carbon element (Augustine et al., 2019). Lv et al. (2019) and Tan et al. (2015) also reported that the destruction of the starch crystalline structure could result in a less intense or partly disappeared diffraction peak. Additionally, Martínez‐Bustos et al. (2007) attributed a partial loss of starch crystallinity to a temperature increase, resulted from mechanical energy conduction or dissipation during grinding process. Therefore, the decrease in intensity and the disappearance of diffraction peaks were mainly attributed to the reduction in crystallite size and the destruction of the crystalline structure.

**Figure 4:**
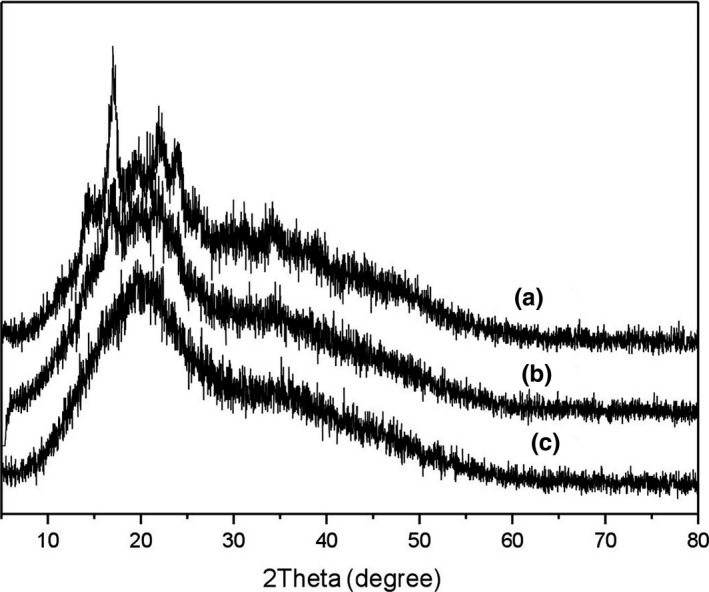
XRD patterns of BFU powders: (a) FP; (b) SP‐I; (c) SP‐II. XRD patterns show the crystalline structure information of BFU powders obtained by different methods. Line (a) corresponds to FP (fine powder, the particle size is 271.95 μm), line (b) corresponds to SP‐I (superfine powder I, the particle size is 45.15 μm), and line (c) corresponds to SP‐II (superfine powder II, the particle size is 154.02 μm)

In general, for all BFU powders, the peak centered at 20° is observed (Figure 4). Normally, amylose–lipid complexes were indicated by a 20° peak, but the complexes were also characterized by two other peaks at 7° and 13° (Godet, Bizot, & Buléon, 1995; Lopez‐Rubio, Flanagan, Gilbert, & Gidley, 2008). Lopez‐Rubio, Flanagan, Gilbert, et al. (2008) assumed that a 20° peak of wheat and rice was mainly due to ordered single amylose helices. Varatharajan et al. (2011) reported that the peak at 20° was due to single left‐handed linear starch chains. From these results, it could be concluded that the structure of starch chains was not destroyed seriously during grinding process.

## 4 DISCUSSION

The grinding process of starch granules was divided into three stages: stress, aggregation, and agglomeration (Zhang et al., 2018). At agglomeration stage, some fragments adhered to the broke starch granule and some fragments agglomerated together. Guzzo et al. (2019) also reported that a strong competition between breakage and agglomeration mechanisms occurred in dry grinding process. The change from breakage to agglomeration can be suggested by large‐size agglomerates. In our research, SP‐I exhibited rough particles with broken edges and similar sizes, and SP‐II exhibited agglomeration of broken particles. This indicates that the breakage was dominant in SP‐I, and the agglomeration was dominant in SP‐II (Zheng et al., 2018). Thus, grinding treatment had a marked mechanochemical effect on BFU powders.

Previous studies reported that the particle size might have a positive or negative correlation effect on powder physicochemical properties; for instance, the particle size might have a negative correlation effect (*p* < .05) on the density characteristics (He et al., 2017; Zhao et al., 2015). In our study, the size was smaller for BFU powders, greater for the angle of repose (from 36.62 to 49.50°) and total alkaloid content (from 0.009% to 0.015%). However, the bulk density, swelling capacity, and ethanol extraction yield in our study were not consistent with the findings due to the performance of SP‐II. This may be potentially related to the occurrence of agglomeration and structure change induced by intense superfine grinding (Guzzo et al., 2019; Huang et al., 2007). Opoczky (1977) also reported that agglomeration had a negative effect on certain properties of the grinding product. Guzzo et al. (2019) reported that the ability of grinding product to hydrate, reactivity, and solubility could be negatively affected because of agglomeration and structural changes induced by high‐energy milling. Hu, Chen, and Ni (2012) and Zhao et al. (2015) indicated that the higher temperature induced by intense grinding would affect the stability of active content in superfine powders. Thus, even though particle size may significantly affect the properties of BFU powder, other phenomena (agglomeration, structure change, etc.) induced by intense grinding also have a significant effect on certain properties.

Decreases in the *D*_90_ size of BFU powder from 271.95 to 45.15 μm significantly increased the ethanol extraction yield, total alkaloid content, and the contents of the peimissine and imperialine. FTIR results indicate that no new functional groups produced after superfine grinding. XRD results indicate the decreased crystallinity after superfine grinding. It was reported that superfine grinding markedly increased the extraction of active ingredients, leading to improved bioavailability and bioactivity in vivo or in vitro (Hu et al., 2012; Tao et al., 2014; Xiao et al., 2017). Therefore, the application of superfine grinding in BFU powders exhibits positive significance in terms of increasing the bioavailability of BFU and saving material resources.

## 5 CONCLUSIONS

In this study, three bulbs of *F. unibracteata* Hsiao et K.C. Hsia (BFU) powders in different sizes were produced via superfine and conventional grinding methods. Particle size and grinding process played an important role in the properties of BFU powders. Specifically, SP‐I exhibited the smallest particle size and the narrowest particle size distribution. Additionally, SP‐I exhibited significantly high total alkaloid content and ethanol extraction yield. This is due to the increase in particle surface area and the breakdown of the cell walls. Although particle size may significantly affect the properties of BFU powder, other phenomena (agglomeration, structure change, etc.) induced by intense grinding also have a significant effect on certain properties. Superfine grinding technology improved utilization efficiency in BFU consumption and displayed the potential to expand the application of BFU powder in the health food and Chinese herb industry.

## CONFLICT OF INTEREST

The authors declare that they do not have any conflict of interest.

## ETHICAL APPROVAL

This study does not involve any human or animal testing.
