# Supplementary material for: Effect of superfine grinding on the physicochemical properties of bulbs of Fritillaria unibracteata Hsiao et K.C. Hsia powder
Source: Food Sci Nutr. 2019 Sep 13;7(11):3527–37. doi: 10.1002/fsn3.1203 (PMC6848833; doi:10.1002/fsn3.1203)
Supplement: Supplementary file 1 [file FSN3-7-3527-s001.docx]

Supplementary information

A certain amount of FP (5.00 g in each tank) was ground with a weight ratio of 20:1 and rotating speed of 350 r/min for 0.25 h, 0.5 h, 1 h, 1.5 h, 2 h, 2.5 h, 3 h, 3.5 h, 4 h, respectively. Fig. S1 shows the sem micrographs of BFU powders with different grinding time, which reveals the effect of mechanical grinding process on the morphology of BFU powders at micrometer level. After 1h of grinding, particle shapes were irregular and the particle edges were broken, indicating that the main effect of the grinding was breakage during this stage. When the grinding time was 2.0 h, there were partial adhesion and agglomeration between the particles. when the grinding time was 3.0 h, the aggregation of broken particles showed a flat structure. when the grinding time was 3.5 h and 4.0 h, the agglomeration structure became more compact. Therefore, during the grinding process, BFU particles experienced breakage, adhesion, and agglomeration.


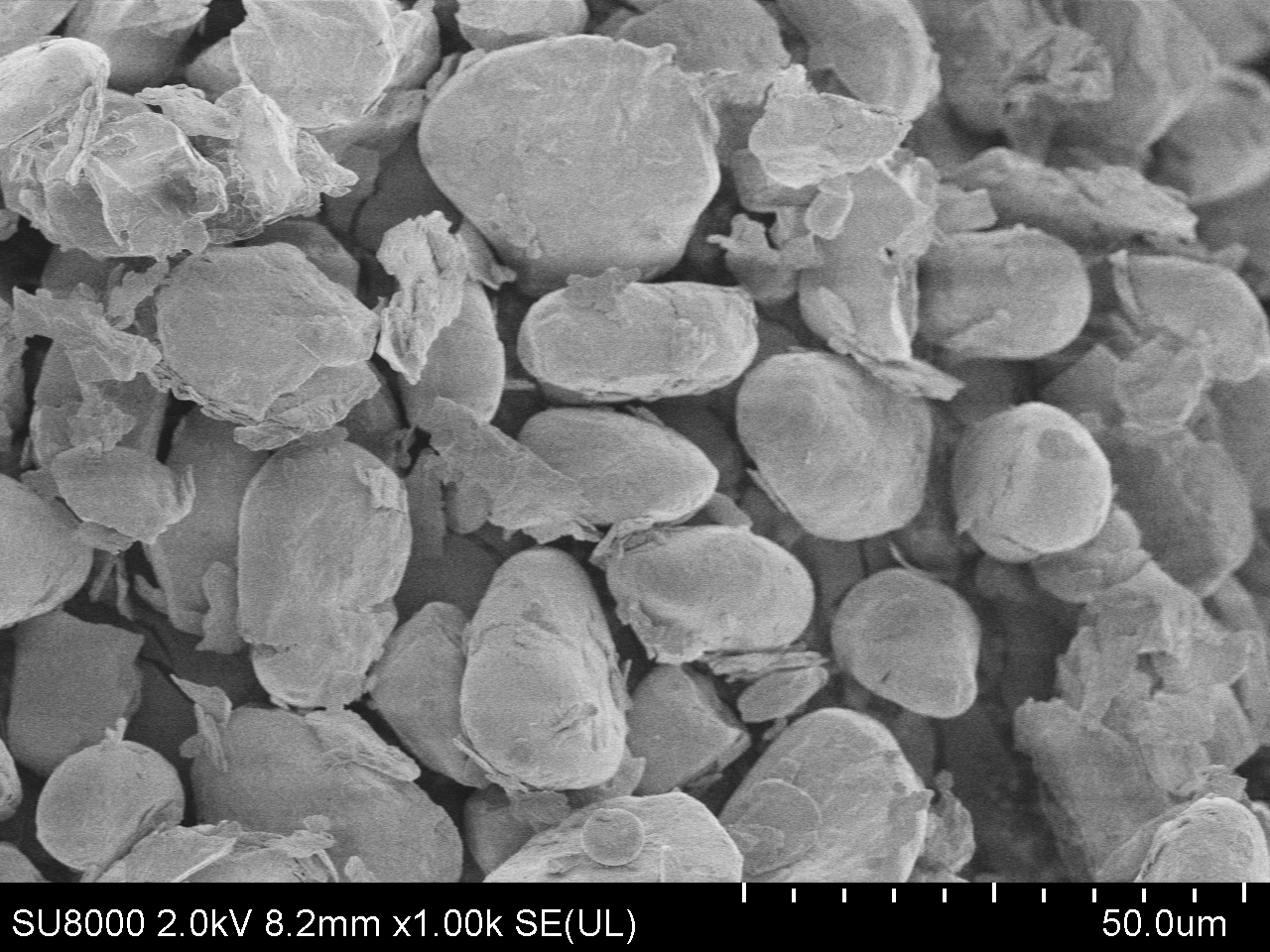

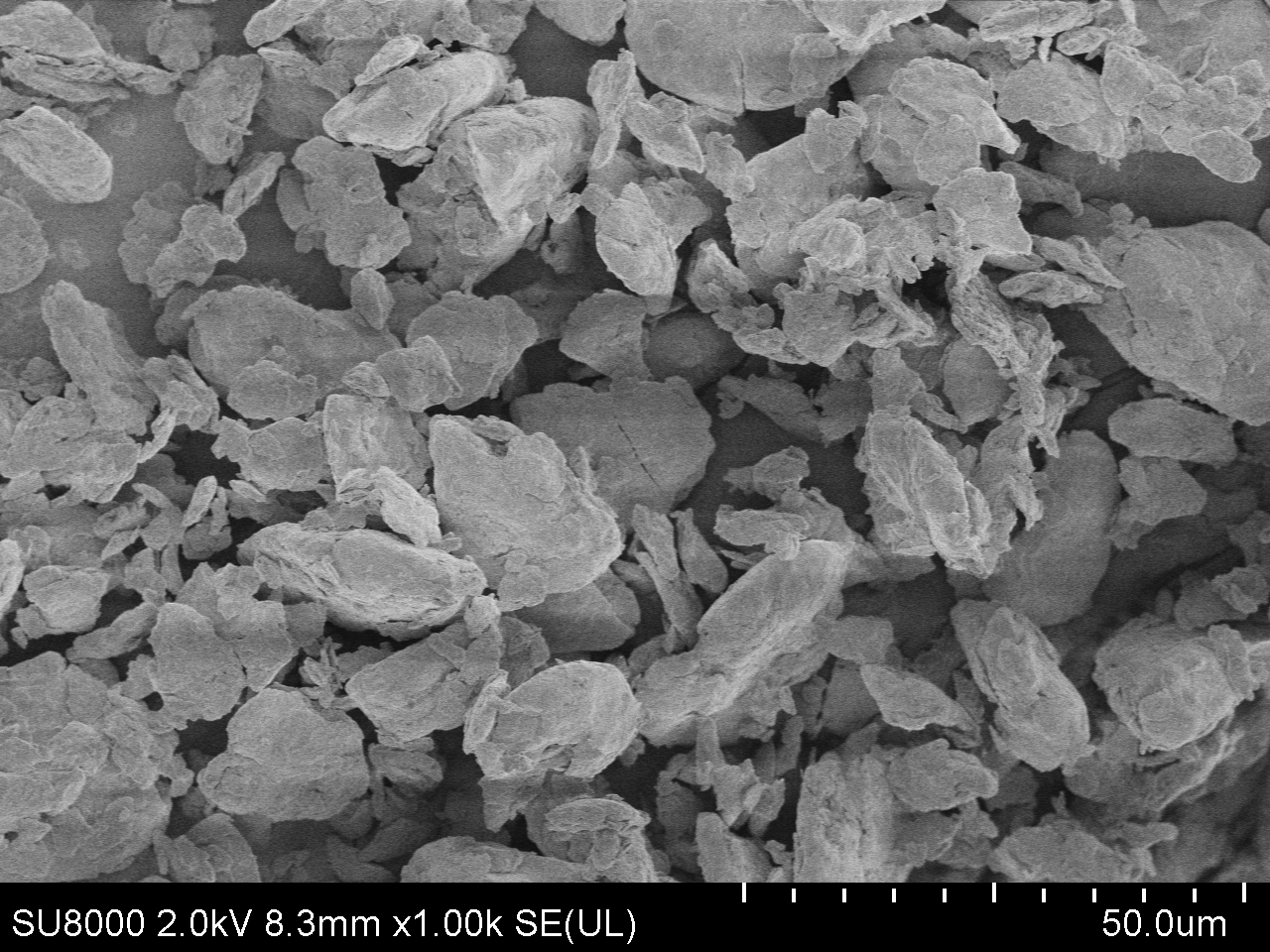

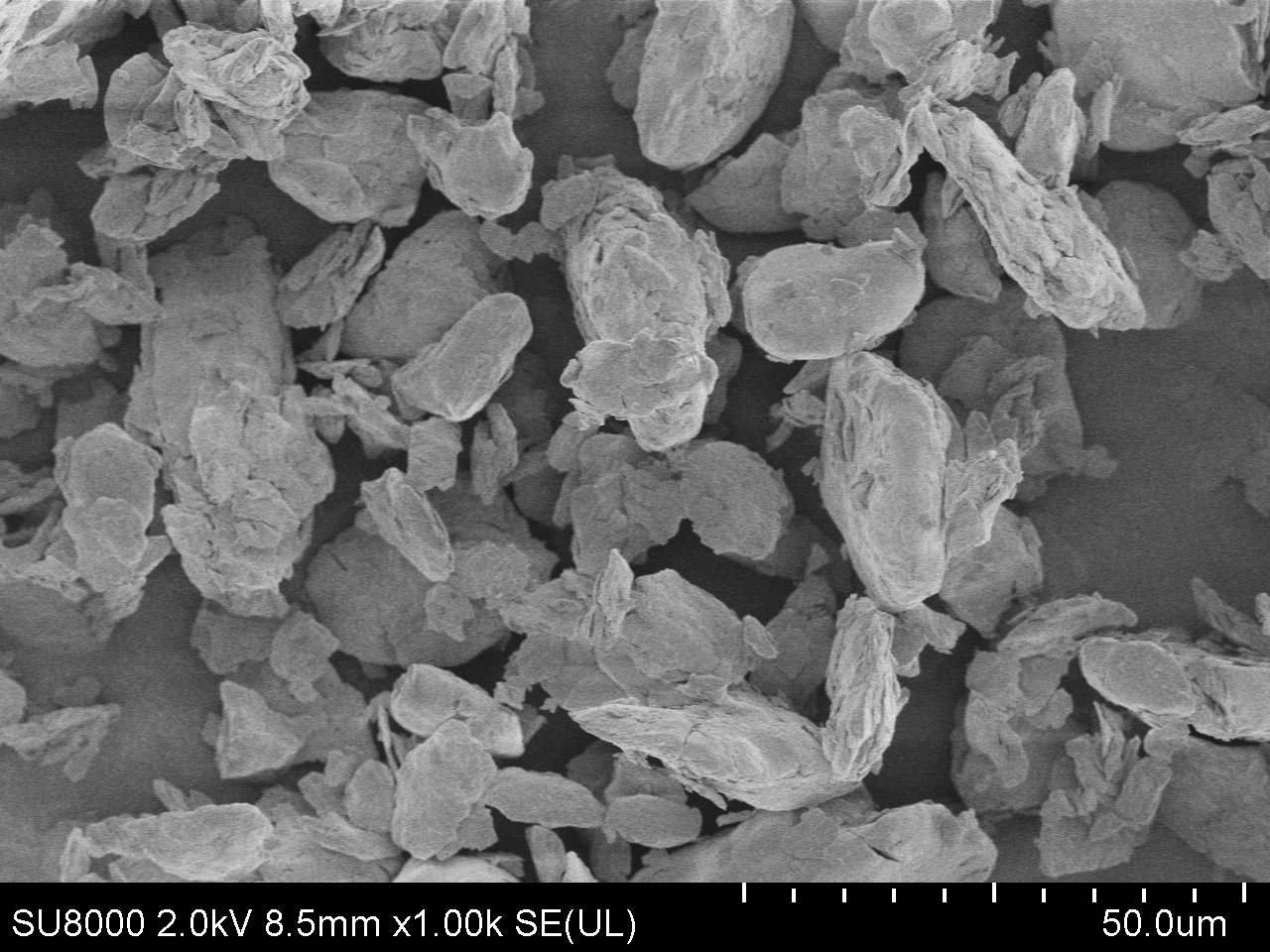


(c)

(b)

(a)


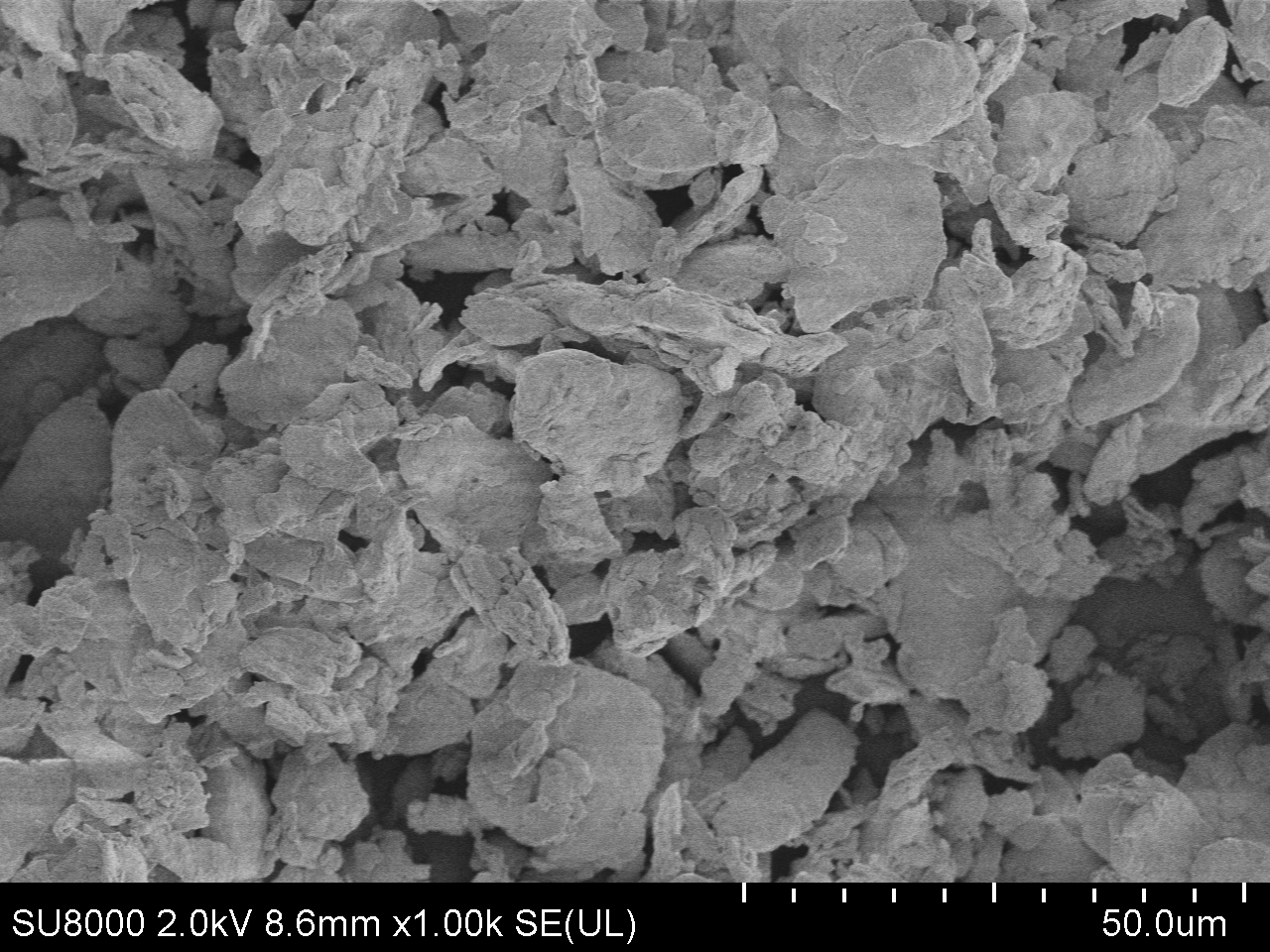

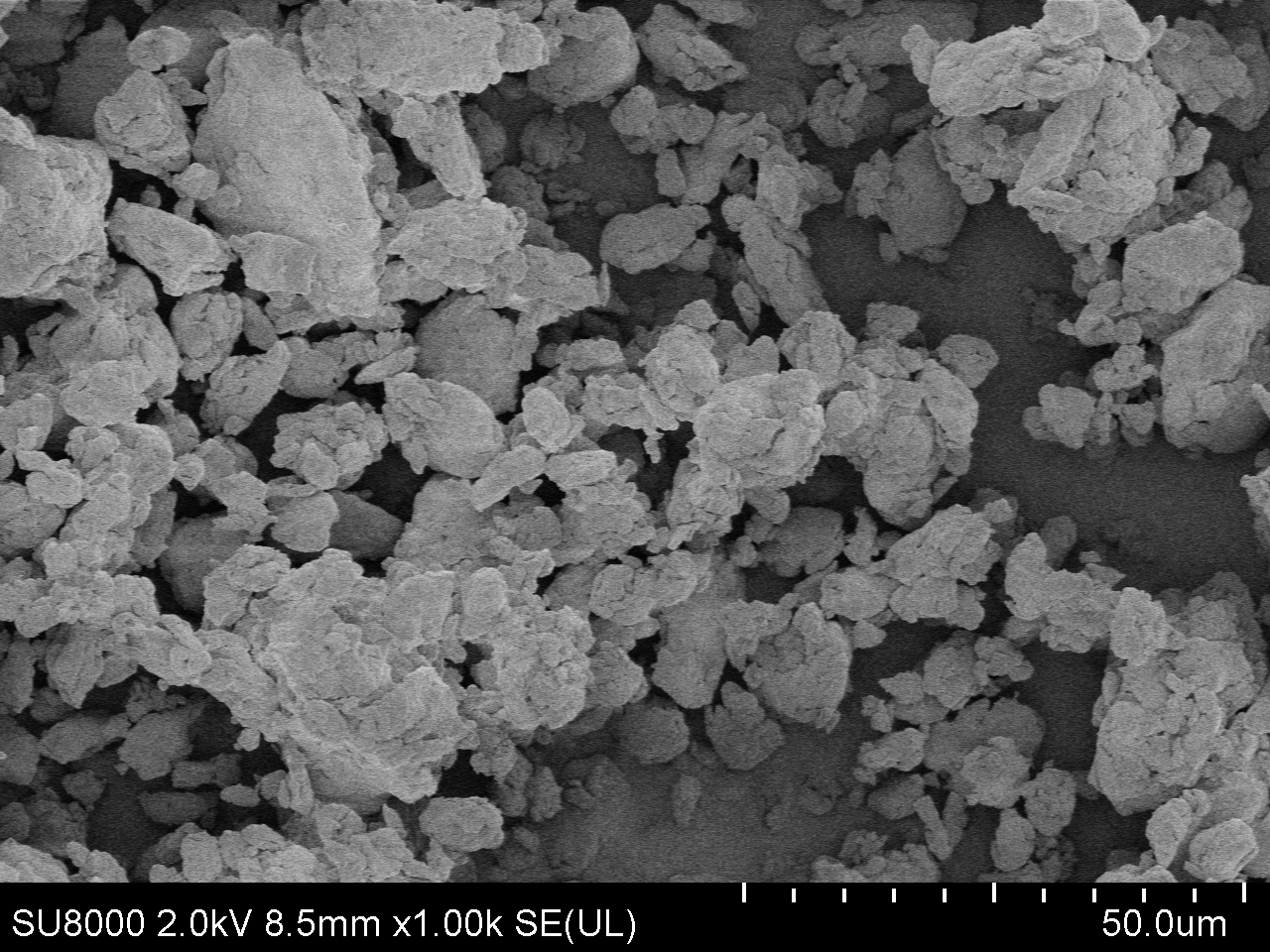

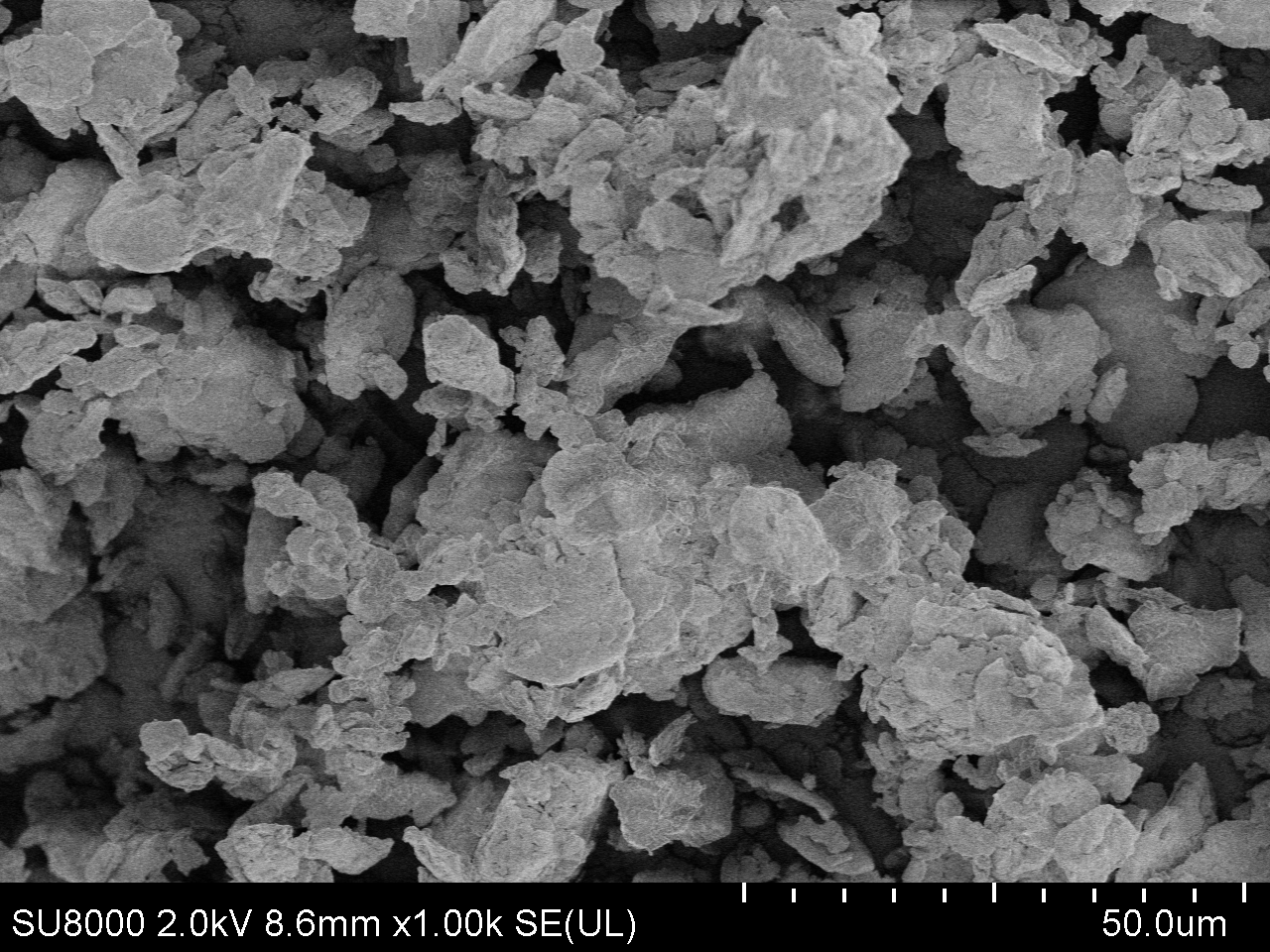


(d)

(e)

(f)


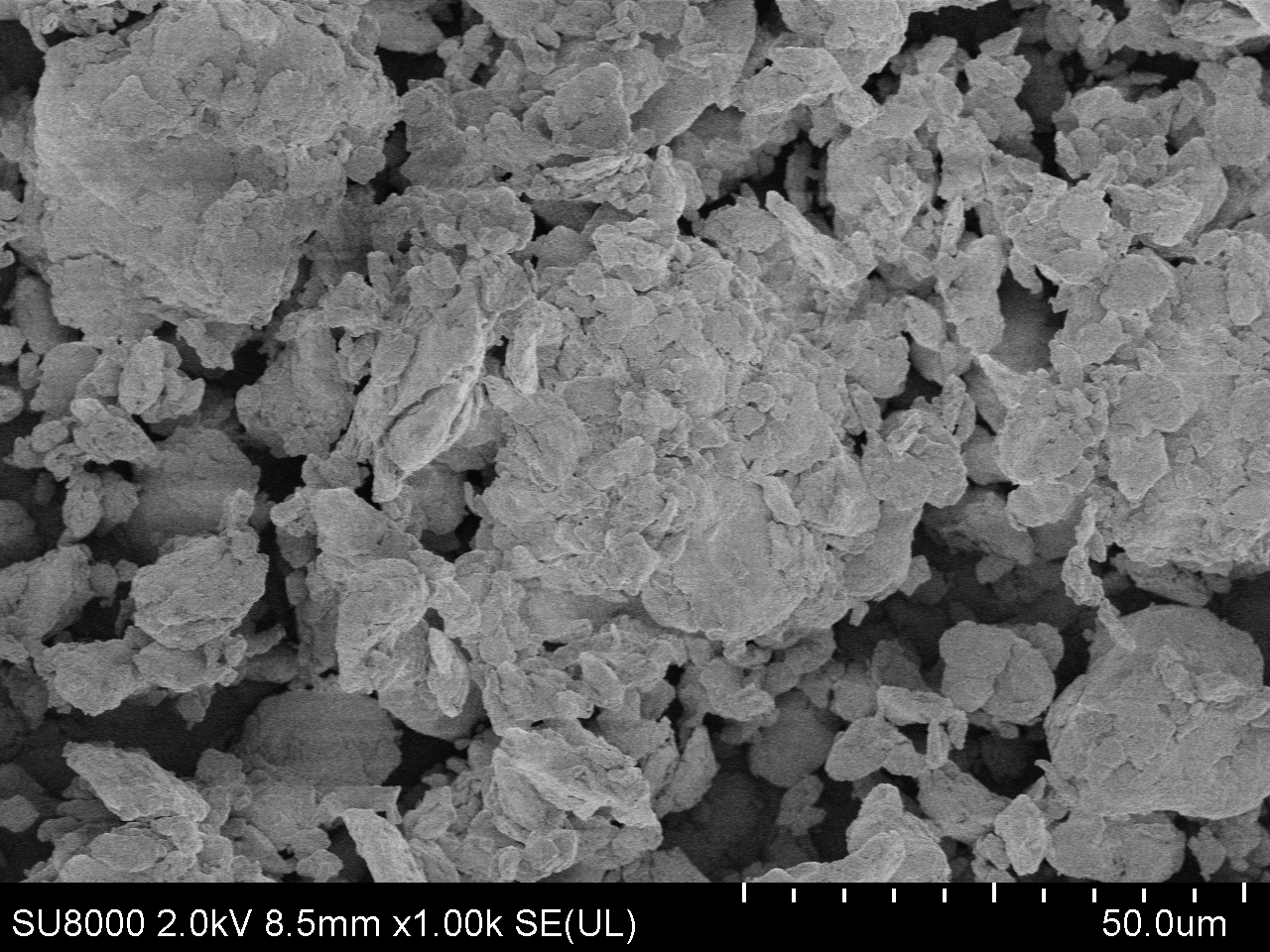

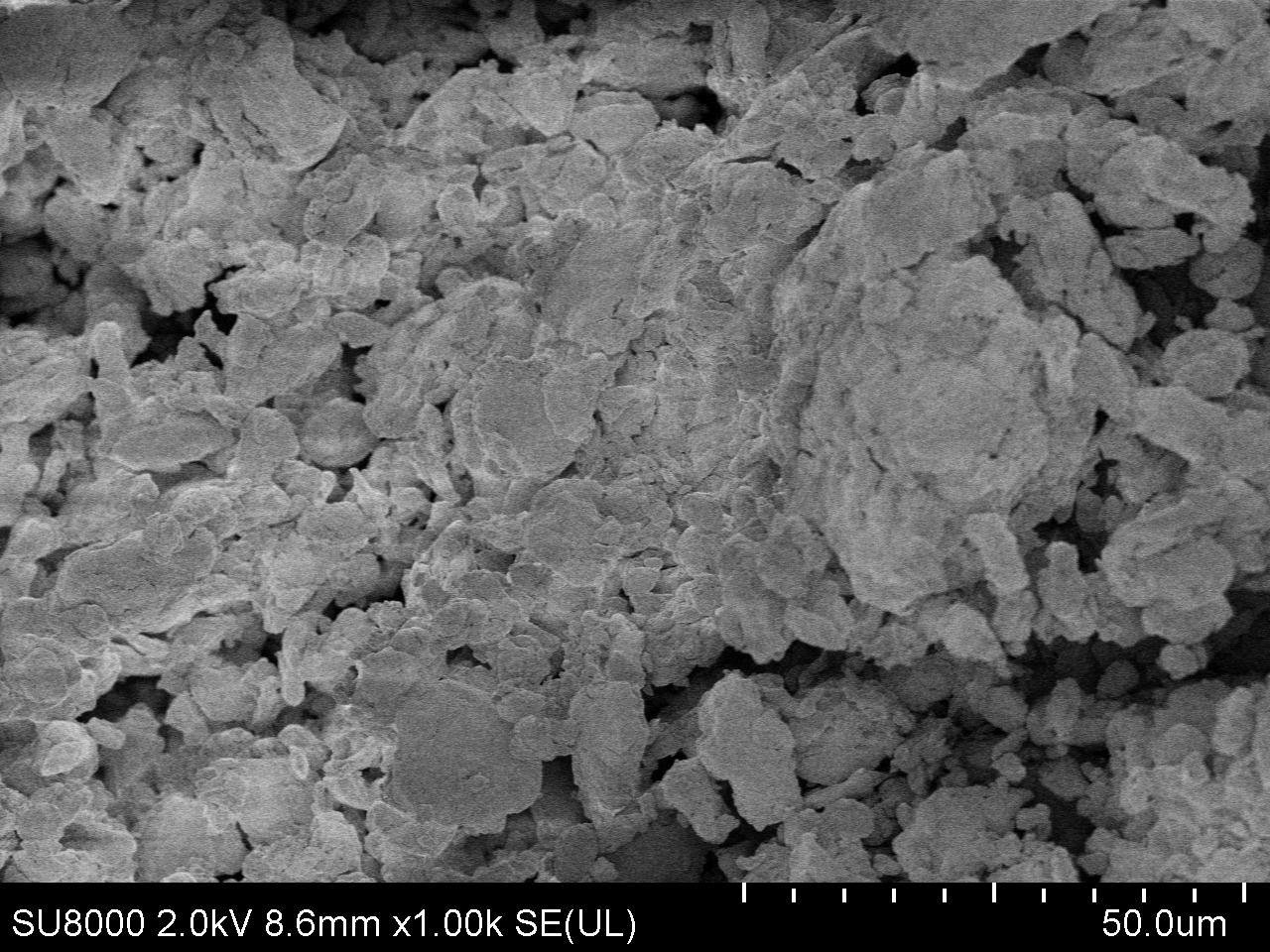

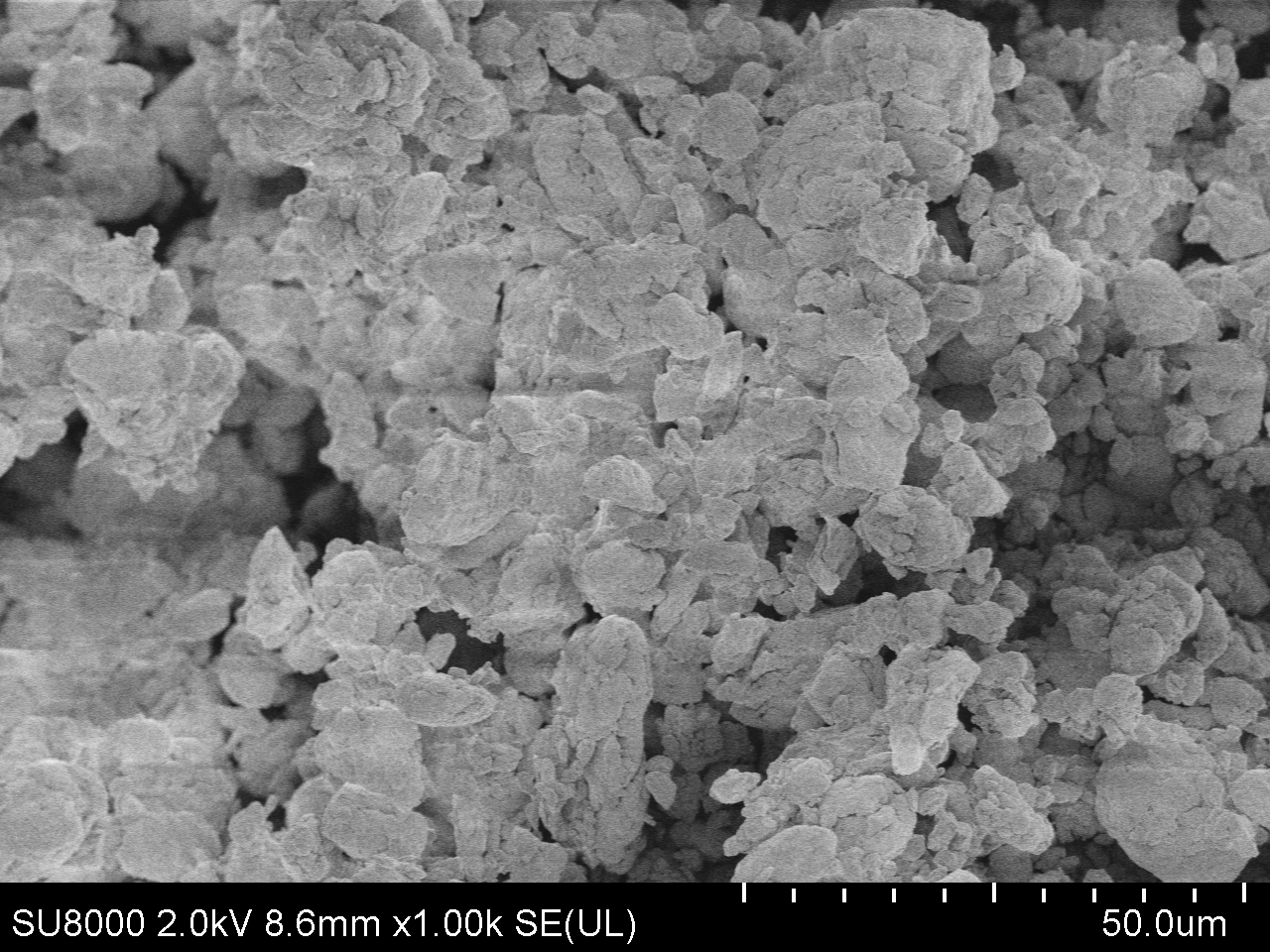


(i)

(h)

(g)

Fig. S1 SEM micrographs of BFU powders with different grinding time:(a)0.25h, (b)0.5h, (c)1h, (d)1.5h, (e)2h, (f)2.5h, (g)3h, (h)3.5h, and (i)4h.
